# Multispectral Palmprint Recognition Using a Quaternion Matrix

**DOI:** 10.3390/s120404633

**Published:** 2012-04-10

**Authors:** Xingpeng Xu, Zhenhua Guo, Changjiang Song, Yafeng Li

**Affiliations:** 1 Bio-Computing Research Center, Harbin Institute of Technology Shenzhen Graduate School, Shenzhen 518055, China; E-Mail: simplexuxingpeng@gmail.com; 2 Graduate School at Shenzhen, Tsinghua University, Shenzhen 518055, China; 3 The Institute of Automation of Heilongjiang Academy of Sciences, Harbin 150090, China; E-Mail: renqi7047@sina.com; 4 Department of Computer Science, Baoji University of Arts and Science, Xi'an 721013, China; E-Mail: liyafeng770729@126.com

**Keywords:** multispectral palmprints, quaternion, PCA, DWT

## Abstract

Palmprints have been widely studied for biometric recognition for many years. Traditionally, a white light source is used for illumination. Recently, multispectral imaging has drawn attention because of its high recognition accuracy. Multispectral palmprint systems can provide more discriminant information under different illuminations in a short time, thus they can achieve better recognition accuracy. Previously, multispectral palmprint images were taken as a kind of multi-modal biometrics, and the fusion scheme on the image level or matching score level was used. However, some spectral information will be lost during image level or matching score level fusion. In this study, we propose a new method for multispectral images based on a quaternion model which could fully utilize the multispectral information. Firstly, multispectral palmprint images captured under red, green, blue and near-infrared (NIR) illuminations were represented by a quaternion matrix, then principal component analysis (PCA) and discrete wavelet transform (DWT) were applied respectively on the matrix to extract palmprint features. After that, Euclidean distance was used to measure the dissimilarity between different features. Finally, the sum of two distances and the nearest neighborhood classifier were employed for recognition decision. Experimental results showed that using the quaternion matrix can achieve a higher recognition rate. Given 3000 test samples from 500 palms, the recognition rate can be as high as 98.83%.

## 1. Introduction

There are two main categories in the automatic traditional personal identification area: token-based methods that rely on personal identification such as driver licenses, passports, and other IDs; and knowledge-based methods that rely on signatures or password-protected access [1]. However, things like keys, ID cards, passports are easy to be lost or stolen, and passwords can be forgotten or lost. In order to avoid these disadvantages, biometrics have becomes more and more popular in personal identification. Compared with traditional authentication methods, biometric features are difficult to be stolen, lost or copied, and altered [1,2]. This makes biometric authentication reliable and efficient in situations where high security is needed. Many biometric features have been studied and used in recent decades, such as fingerprints [3,4], palmprints [5–8], iris images [9–12], human faces [13–16] and finger-knuckle-prints [17].

Among these features, palmprints are widely studied [5–8], as they have many merits, being user-friendly, low cost, robust, and highly reliable [5]. Traditional palmprint recognition systems use images acquired using a single source of illumination, which may cause different palms to have similar appearance due to the limited information.

To address the issue of limited information, we have applied multispectral imaging which can provide several images of the same scene with different illuminations for enhanced biometrics applications that include face recognition [14] and iris recognition [12]. Some pioneering work on multispectral palmprints has also been proposed. Rowe *et al.* [18] designed a prototype of a whole-hand multispectral imaging system. Zhang *et al.* [19] developed a fast multispectral palmprint prototype system. Hao *et al.* [20] proposed a new touchless multispectral palmprint system which is different from those in [18] and [19], but similar to the typical iris recognition system, where the user does not need to touch the sensor. These works regarded multispectral palmprint images as a kind of multi-modal biometrics and used fusion schemes on different levels, such as image level [20] and matching score level [18,19]. However, some useful information is lost in the image fusion [21] while correlation between different images is neglected in matching score level fusion as each image is compared to each spectrum individually. To fully utilize multispectral information, a new multispectral palmprint recognition method based on a quaternion matrix was proposed in this study. Our previous work [22] showed the effectiveness of the global quaternion model for palmprint recognition, but the local quaternion feature was not explored. To this end, this study proposes a method based on quaternion representation by incorporating both local and global features.

The concept of quaternion was first proposed in 1843 by the Irish mathematician William Rowan Hamilton [23]. As a quaternion model could represent several bands or matrixes in one complex matrix without losing information, it has been used in color image processing [24,25] and multi-feature processing [26]. In this work, multispectral palmprint images captured by red, green, blue and near-infrared (NIR) [14] are represented by a quaternion matrix first, then principal component analysis (PCA) and discrete wavelet transform (DWT) were applied on the matrix to extract palmprint features individually. After that, Euclidean distance was used to measure dissimilarity between different features. Finally, matching score fusion and the nearest neighborhood classifier were employed for recognition. Because the quaternion matrix fully utilizes the information of multispectral images, higher recognition accuracy is expected in comparison to the traditional fusion schemes.

The rest of the paper is organized as follows: a multispectral imaging device is briefly described in Section 2. The proposed method is outlined in Section 3. Experimental results are reported in Section 4. Finally, the conclusions are given in Section 5.

## 2. Multispectral Imaging Sensor

**Figure 1.**
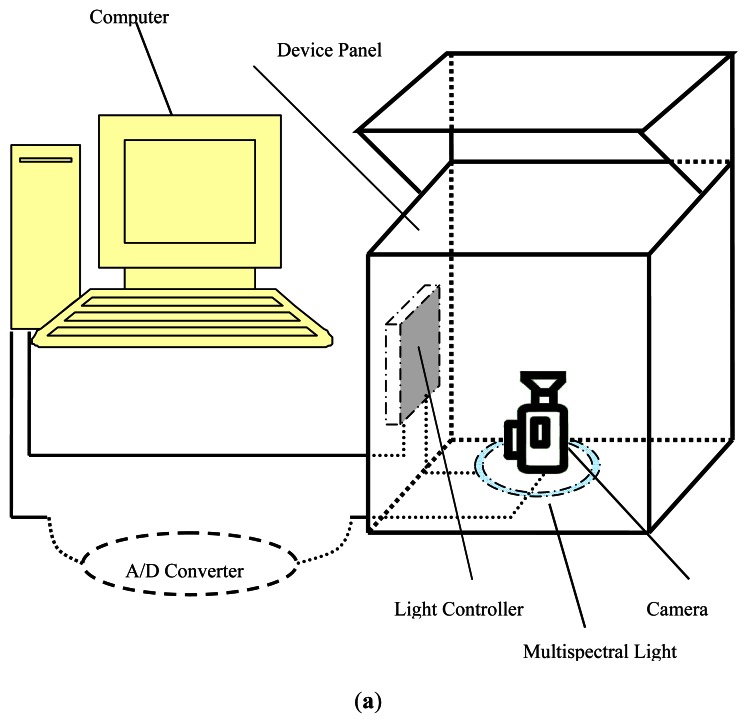

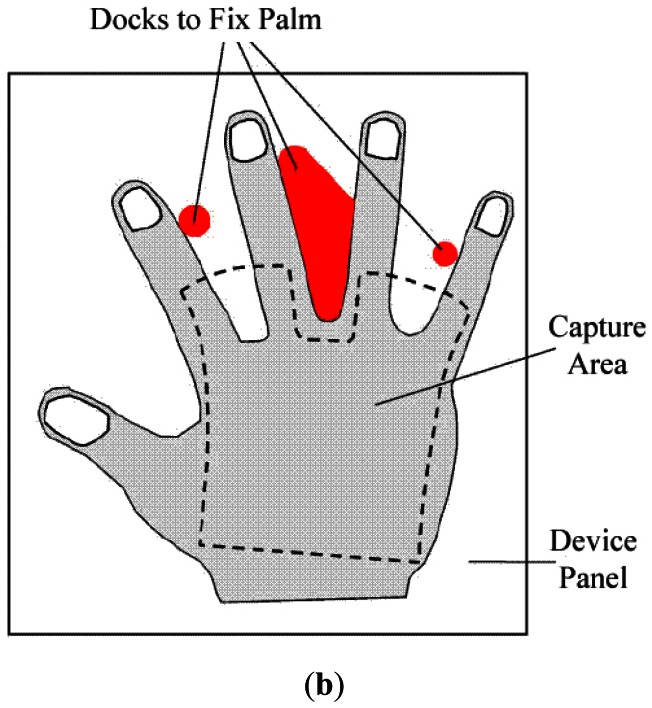
(**a**) The structure of the multispectral palmprint imaging sensor. (**b**) Device panel and how the palm of the hand is situated.

Figure 1 shows the structure of the designed multispectral palmprint imaging sensor and how the palm is situated.

**Figure 2.**
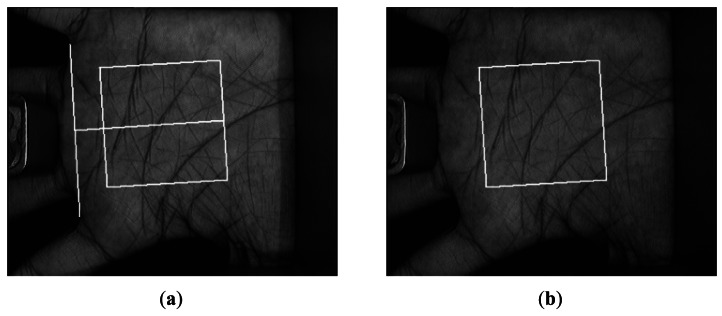

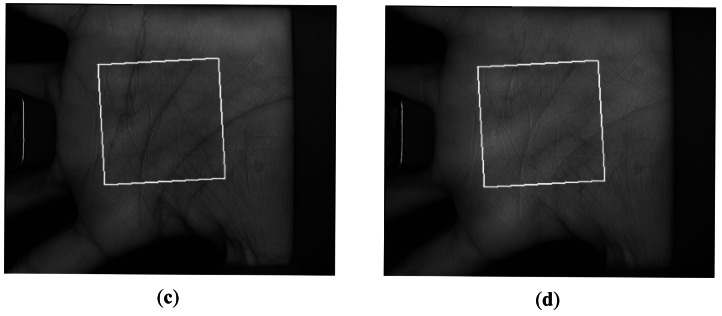
A typical multispectral palmprint sample. (**a**) Blue; (**b**) Green; (**c**) Red; (**d**) NIR. The white square is the region of interest (ROI) of the image.

The key components of the multispectral palmprint imaging device include a CCD camera, lens, and A/D converter. To ensure a semi-closed environment, the box containing the camera is made of opaque plastic and the central part of the device panel is hollow. The multispectral images were captured with four different wavelengths: NIR (880 nm), red (660 nm), green (525 nm) and blue (470 nm) [27]. These wavelengths were chosen because different lights can penetrate different skin layers and enhance different features [28]. The system can capture the palmprint images at a resolution of 352 × 288. During the image capture process, users are asked to put the palm on a platform. The four different palmprint images with the resolution lower than 100 DPI (dot per inch) can be captured in a short time (less than 1 second) [19]. Figure 2 shows a typical multispectral palmprint sample.

## 3. The Framework of the Proposed Method

**Figure 3.**
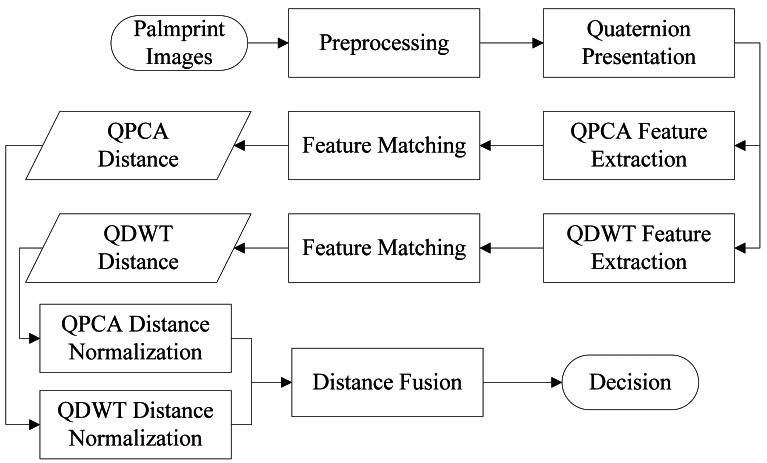
The framework of the proposed method.

Figure 3 shows the whole framework of the proposed method. It includes four key steps: preprocessing, quaternion representation, feature extraction and matching.

### 3.1. Preprocessing

**Figure 4.**
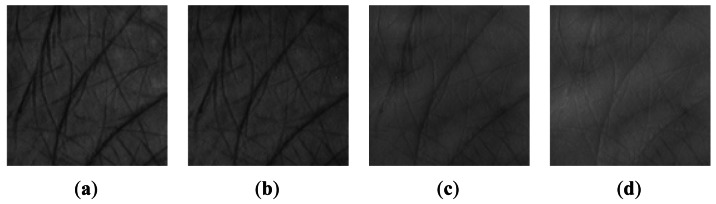
ROI of Figure 2. (**a**) Blue; (**b**) Green; (**c**) Red; (**d**) NIR.

Before extracting palmprint features, a region of interest (ROI) of a palmprint image is selected first. A coordinate system to reduce rotation and translation effects is built from the given image and then a 128 × 128 ROI [5] is cropped from the whole image. Figure 4 illustrates the extracted ROI from Figure 2 by the method proposed in [5]. After that, histogram equalization is used to remove global intensity influence.

### 3.2. Quaternion Representation

To fully utilize the information of the multispectral palmrpint images, the multispectral palmprint images are represented by a quaternion matrix. In mathematics, quaternions are a noncommutative number system. A quaternion is a linear combination of a real scalar and three imaginary units:

(1) q=a+bi+cj+dk

where *i, j, k* are three imaginary units. Their relationship is *i*^2^ = *j*^2^ = *k*^2^ = *ijk* = −1.

The conjugate of a quaternion is: q*=*a-bi-cj-dk*, and the norm of a quaternion is:

(2) $$|q|=\sqrt{qq^{*}}=\sqrt{a^{2}+b^{2}+c^{2}+d^{2}}$$

*I*(x, *y*), *R*(x, *y*), *G*(x, *y*), and *B*(x, *y*) are used to represent four ROI images by NIR, red, green and blue illuminations, respectively. After preprocessing, a quaternion matrix, *Q*(*x, y*), is constructed by these four ROI images:

(3) Q(x,y)=I(x,y)+R(x,y)⋅i+G(x,y)⋅j+B(x,y)⋅k

In Equation (3), each pixel of *Q*(*x, y*) is represented by a 4D point. If there are less than four illuminations, we can still construct a quaternion matrix by replacing the missing illumination with a zero-matrix. This can also give good results, as shown in Section 4.

### 3.3. Feature Extraction

In this study, two kinds of features are extracted from a quaternion matrix, quaternion PCA (QPCA) [24] which captures global appearance of multispectral palmprint images, and quaternion DWT (QDWT) [29] which represents local texture information.

#### 3.3.1. QPCA Feature Extraction

**Figure 5.**
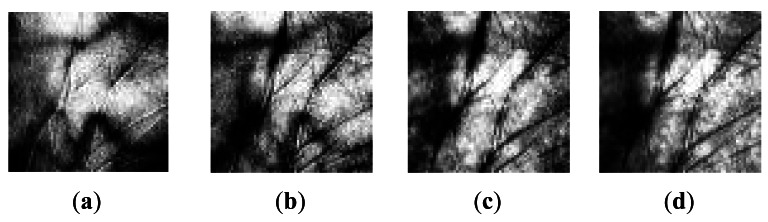
The ROIs of multispectral palmprint images under 4 different kinds of illuminations after the preprocessing and downsampling. (**a**) NIR. (**b**) Red. (**c**) Green. (**d**) Blue.

**Figure 6.**
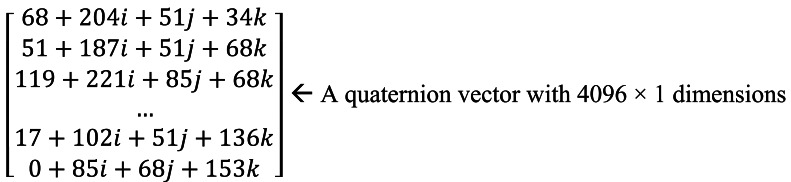
A quaternion vector sample built using the input image in Figure 5.

To speed up computation and reduce memory cost [8], the ROIs are downsampled to 64 × 64 as shown in Figure 5. In the future, instead of down-sampling, we will investigate advanced methods, such as incremental multilinear PCA [30]. The quaternion matrix is first converted to a quaternion vector recorded by row. Figure 6 illustrates a quaternion vector sample built by Figure 5. Then a new matrix for learning projection matrix is built by *n* training samples. *S_m_*_×_*_n_* = [*Q*_1_ *Q*_2_…*Q_n_*]. Each column is a quaternion vector of one multispectral palmprint sample and its length is *m* (64 × 64).

Given the matrix *S_m_*_×_*_n_*, its covariance matrix is *C_m_*_×_*_m_*. However, computing the eigenvectors and the eigenvalues of the matrix *C_m_*_×_*_m_* is an intractable task for such a high dimension. In fact, the number of samples *n* is relatively small, thus it is easy to calculate eigenvectors and eigenvalues of matrix *C_n_*_×_*_n_*:

(4) $$C_{n\times n}=\frac{1}{n-1}E^{T*}E$$

(5) $$E=S-\bar{S}$$

(6) $$\bar{S(x,y)}=\frac{1}{n}\sum_{l=1}^{n}S(x,l),x=1\ldots m,y=1,\ldots ,n$$

where T*is the conjugate-transposition operator for a quaternion matrix. *C_n_*_×_*_n_* is a Hermitian matrix, so through Householder transformations [24,31], it could be tridiagonalized to obtain, *B*, which is a real tridiagonal symmetric matrix, and *P*, which is the product of Householder matrices used to compute matrix:

(7) $$C_{n\times n}=P^{T*}BP$$

Matrix *B* is a real matrix, so eigenvectors of *B* are easily calculated. *V_B_* is used to represent the eigenvectors matrix of *B*, each column of *V_B_* is an eigenvector of matrix *B*. Thus eigenvectors of *C_n_*_×_*_n_* can be calculated as:

(8) $$V_{C}=P^{T*}V_{B}$$

Eigenvalues of *C_n_*_×_*_n_* are eigenvalues of matrix *B* as well. *D_c_* is used to represent eigenvalues of *C_n_*_×_*_n_*. Now we have the eigenvectors and the eigenvalues of matrix *C_n_*_×_*_n_*. Finally eigenvalues and eigenvectors of matrix *C_m_*_×_*_m_* is computed by:

(9) $$V=EV_{C}$$

(10) $$D=\frac{n-1}{m-1}D_{C}$$

where *V* consists of eigenvectors of *C_m_*_×_*_m_*, and *D* consists of eigenvalues of *C_m_*_×_*_m_*.

Eigenvalues *D* are sorted in descending order, and an energy ratio is defined as:

(11) $$\text{ratio}=\sum_{x=1}^{p}D_{x}/\sum_{x=1}^{n}D_{x}\times 100\%$$

where *D_x_* is the *x*^th^ eigenvalue. Given an energy ratio, the number of the eigenvalues *p* can be calculated by using Equation (11). The projection matrix *P̂* is constructed by the first *p* eigenvectors in the matrix *V*. In this work, the energy ratio is set to 90% for a balance between accuracy and feature dimension.

For an input quaternion sample *s*, the QPCA feature *f_QPCA_* is computed as:

(12) $$f_{\text{QPCA}}={\hat{P}}^{T*}s$$

#### 3.3.2. QDWT Feature Extraction

After one scale decomposition, four groups of coefficients will be generated [27,32] by quaternion discrete wavelet. These coefficients include one group of approximation coefficients and three groups of detail coefficients on three directions (horizontal, vertical and diagonal). Here, only approximation coefficients are used for texture feature, as detail coefficients are sensitive to noise.

Given a quaternion sample *Q* = *I* + *R* · *i* + *G* · *j* + *B* · *k*, the approximation coefficients *coef^Q^* are:

(13) $${\text{coef}}^{Q}={\text{coef}}^{I}+{\text{coef}}^{R}\cdot i+{\text{coef}}^{G}\cdot j+{\text{coef}}^{B}\cdot k$$

(14) $${\text{coef}}^{I}=(I⊗g_{1})⊗g_{1}+(G⊗g_{2})⊗g_{1}+(G⊗g_{1})⊗g_{2}-(I⊗g_{2})⊗g_{2}$$

(15) $${\text{coef}}^{R}=(R⊗g_{1})⊗g_{1}+(B⊗g_{2})⊗g_{1}+(B⊗g_{1})⊗g_{2}-(R⊗g_{2})⊗g_{2}$$

(16) $${\text{coef}}^{G}=-(I⊗g_{1})⊗g_{2}-(G⊗g_{2})⊗g_{2}-(G⊗g_{2})⊗g_{1}+(I⊗g_{1})⊗g_{1}$$

(17) $${\text{coef}}^{B}=-(R⊗g_{1})⊗g_{2}-(B⊗g_{2})⊗g_{2}-(B⊗g_{2})⊗g_{1}+(R⊗g_{1})⊗g_{1}$$

where ⊗ is the convolution operator, *g*_1_ and *g*_2_ are two filters specifically designed by He and Li for QDWT [29]:

(18) $$g_{1}=[\begin{array}{ccccccccccccc} 0 & 0 & 0 & 0 & 0 & \frac{1}{4\sqrt{2}} & 0 & \frac{3}{4\sqrt{2}} & \frac{1}{\sqrt{2}} & 0 & 0 & 0 & 0 \end{array}]/\sqrt{2}$$

(19) $$g_{2}=[\begin{array}{ccccccccccccc} 0 & 0 & 0 & 0 & 0 & 0 & 0 & \frac{-\sqrt{3}}{4\sqrt{2}} & 0 & \frac{\sqrt{3}}{4\sqrt{2}} & 0 & 0 & 0 \end{array}]/\sqrt{2}$$

To get stable features, the quaternion matrix is divided into non-overlapping blocks. Each block is with size of z × z. The *z* is empirically set as 5 to balance between accuracy and feature dimension.

For each block:

(20) $$\text{bloc}k_{z\times z}={\text{coef}}_{z\times z}^{I}+{\text{coef}}_{z\times z}^{R}\cdot i+{\text{coef}}_{z\times z}^{G}\cdot j+{\text{coef}}_{z\times z}^{B}\cdot k$$

The standard deviation of this block is a quaternion constructed by the standard deviation of each band in the block.

(21) $$\sigma_{l}=\sigma ({\text{coef}}_{z\times z}^{I})+\sigma ({\text{coef}}_{z\times z}^{R})\cdot i+\sigma ({\text{coef}}_{z\times z}^{G})\cdot j+\sigma ({\text{coef}}_{z\times z}^{B})\cdot k$$

where *σ_l_* is the standard deviation of the *l*^th^ block. Then a quaternion vector is built by concatenating all standard deviations and this quaternion vector is used as the QDWT feature:

(22) $$f_{\text{QDWT}}=[\sigma_{1},\sigma_{2},\ldots ,\sigma_{h}]$$

where *h* is the number of the blocks used.

#### 3.3.3. Feature Matching

Euclidean distance between two quaternions *p* and *q* is defined as:

(23) d(p,q)=|p−q|

After feature extraction, one multispectral palmrpint sample has two different features, one QPCA feature *f_QPCA_* and one QDWT feature *f_QDWT_*. Thus, two different distances, one QPCA distance and one QDWT distance are gotten. The QPCA distance *d_QPCA_* is the Euclidean distance between two QPCA features $f_{\text{QPCA}}^{u}$ and $f_{\text{QPCA}}^{v}$ of two given samples: *s^u^* and *s^v^*, while the QDWT distance *d_QDWT_* is the Euclidean distance between two QDWT features $f_{\text{QDWT}}^{u}$ and $f_{\text{QDWT}}^{v}$:

(24) $$d_{\text{QPCA}}=d(f_{\text{QPCA}}^{u},f_{\text{QPCA}}^{v})$$

(25) $$d_{\text{QDWT}}=d(f_{\text{QDWT}}^{u},f_{\text{QDWT}}^{v})$$

The final distance between two samples is the weighted fusion of the QPCA distance and the QDWT distance. Before fusion, we need to normalize QPCA and QDWT distances by dividing each of these distances by their respective standard deviation of the distances between the training samples, as expressed below [33]:

(26) $${\bar{d}}_{\text{QPCA}}=\frac{d_{\text{QPCA}}}{\sigma_{\text{QPCA}}^{\text{Training}}}$$

(27) $${\bar{d}}_{\text{QPCA}}=\frac{d_{\text{QPCA}}}{\sigma_{\text{QPCA}}^{\text{Training}}}$$

where $\sigma_{\text{QPCA}}^{\text{Training}}$ and $\sigma_{\text{QDWT}}^{\text{Training}}$ are the standard deviation of the distance between the training samples. After the normalization, the distance between two given samples can be calculated:

(28) $$d={\bar{d}}_{\text{QPCA}}\cdot w_{\text{QPCA}}+{\bar{d}}_{\text{QDWT}}\cdot w_{\text{QDWT}}$$

where *w_QPCA_* is the weight of the QPCA distance and *w_DWT_*_=_ 1-*w_QPCA_* is the weight of the QDWT distance. We can adjust the distance by adjusting the weights to achieve higher recognition accuracy. In this work, the weights of QPCA distance and the QDWT distance are set as 0.6 and 0.4 empirically.

## 4. Experimental Results

The experiments are based on a large multispectral palmprint database [19]. The nearest neighborhood classifier is used, and recognition accuracy is computed for performance evaluation.

### 4.1. Multispectral Palmprint Database

The database consists of 500 different palms, and each palm was sampled 12 times in two sessions with a time interval of about 5–15 days. In each session, the subjects were asked to provide six groups of images. Each group contained four images under four different illuminations, so there were 6,000 groups of palmprint images [19]. The 3,000 groups of palmprint images captured in the first session were used as the training samples, and the remaining samples were used as the test samples.

### 4.2. Recognition Accuracy

**Figure 7.**
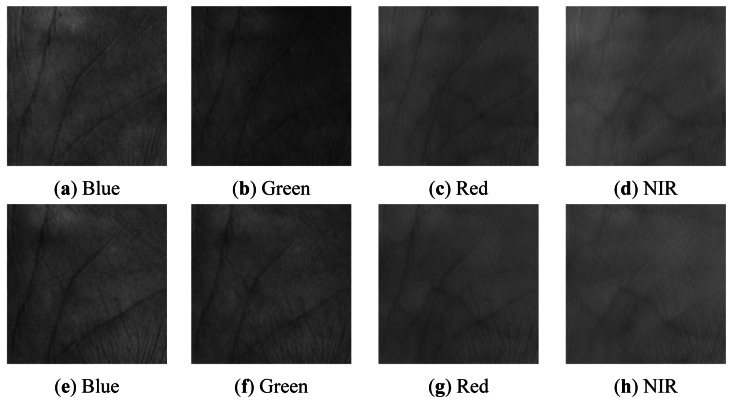
A pair of multispectral palmprint images from the same palm but falsely recognized. (**a-d**) are four images of one instance, and (**e-h**) are four images of another instance of different time.

Recognition accuracy is obtained by matching each palmprint sample in the test set with all the samples in the training set. Table 1 shows the recognition accuracy of different methods. From Table 1, we can see that quaternion representation can improve recognition accuracy significantly comparing to the single illumination. As quaternion representation keeps all the information in feature extraction, it could get better results than image level fusion and matching score level fusion. The combination of QPCA and QDWT can further improve the recognition accuracy. Figure 7 shows a pair of multispectral palmprint images which are collected from the same palm but classified wrongly. As shown in Figure 7, ROI extraction influences somewhat the recognition of the proposed method. ROI extraction will therefore be the focus of our future work to improve the recognition accuracy.

### 4.3. Special Arrangement When the Number of Illuminations Is Less Than 4

To evaluate the performance of our method in the situation with less than four illuminations, we replace some bands with a zero-matrix. Table 2 shows the recognition accuracy of different situations using QDWT. The band marked 1 means that this band is used in the quaternion matrix and the band marked 0 means that we use a zero-matrix to replace this band in the matrix.

From Table 2, we can find that the recognition accuracy is still better than single illumination when we replace some of the bands with zero matrixes. The finding is similar to the result using QPCA. It validates that quaternion representation is applicable for the situation with less than four feature bands. However, the proposed method is limited to a maximum of four bands. How to extend the quaternion representation for more bands will be a topic for our future work.

Table 3 illustrates the correlation between different spectra. Several findings could be found from Tables 2 and 3. First, as shown in Table 3, when the wavelength difference between two spectra increases, the correlation of palmprint images becomes lower. For example, the correlation between Blue and Green is 0.7421, while the correlation between Blue and NIR is only 0.4487. Second, when the correlation between two spectra is low, the palmprint images by these two spectra contain more complementary information, thus the accuracy improvement by their fusion is more significant. Thus, QDWT of NIR and Blue could reach 98.13%, while the QDWT of Green and Blue is only 94.87%. Third, a quaternion model is an efficient method to utilize the information of multispectral palmprint images, as the best accuracy using three or two spectra is 98.13%, which is smaller than using all of the spectra (98.50%).

### 4.4. Speed

The experiment is implemented using Matlab 7.0 on a PC with Windows XP (x64), Xeon 5160 CPU (3.0GHz), and 4-GB RAM. The execution time for preprocessing, feature extraction and feature matching is listed in Table 4. As shown in Table 4, the proposed method is fast enough for real time applications.

## 5. Conclusions

In this paper and to fully utilize the information of multispectral palmprint images, to the best of our knowledge, a quaternion model is employed for multispectral biometrics for the first time. QPCA is proposed for representing global features while QDWT is designed for extracting local features. Their fusion could achieve 98.83% recognition accuracy for 500 palms. The experimental results show that the proposed method is good enough for real applications and the quaternion model is an effective and efficient technique for multispectral biometrics. The special arrangement has shown that the quaternion matrix is still effective for multispectral palmprint in the situation with less than four illuminations.

In the future, we will try to apply the proposed method to other multispectral biometrics, such as face and iris recognition. We will also explore advanced feature extraction methods on the quaternion matrix, such as the kernel method [34]. How to represent multispectral palmprint images with more than four bands is another research direction, and finding the similarity between palms as a quantitative measure [35] will be explored also.

## Figures and Tables

**Table 1.** Recognition Accuracy.

**Experiments**	**Recognition Accuracy**
NIR PCA	94.60%
Red PCA	96.30%
Green PCA	93.47%
Blue PCA	93.47%
Image level fusion by PCA	95.17%
Matching score level fusion by PCA	98.07%
**QPCA**	**98.13%**
NIR DWT	94.60%
Red DWT	95.20%
Green DWT	93.50%
Blue DWT	93.83%
Image level fusion by DWT	96.60%
Matching level score fusion by DWT	98.00%
**QDWT**	**98.50%**
**QPCA+QDWT**	**98.83%**

**Table 2.** Recognition accuracy of different situations using QDWT.

**NIR**	**Red**	**Green**	**Blue**	**Recognition Accuracy**
1	1	0	0	97.17%
1	0	1	0	98.10%
1	0	0	1	98.13%
0	1	1	0	97.23%
0	1	0	1	97.33%
0	0	1	1	94.87%
1	1	1	0	98.03%
1	1	0	1	97.90%
1	0	1	1	98.13%
0	1	1	1	97.10%

**Table 3.** Image correlation between different spectra.

	**NIR**	**Red**	**Green**	**Blue**
**NIR**	1	-	-	-
**Red**	0.7470	1	-	-
**Green**	0.3690	0.5060	1	-
**Blue**	0.4487	0.6829	0.7421	1

**Table 4.** Execution Time.

	**Average Time (ms)**
Preprocessing	20
QPCA feature extraction	46
QDWT feature extraction	547
QPCA feature matching	0.42
QDWT feature matching	0.43
